# Disparity Changes in 370 Ma Devonian Fossils: The Signature of Ecological Dynamics?

**DOI:** 10.1371/journal.pone.0036230

**Published:** 2012-04-27

**Authors:** Catherine Girard, Sabrina Renaud

**Affiliations:** 1 Institut des Sciences de l'Evolution, Université Montpellier 2, CNRS, Montpellier, France; 2 Laboratoire de Biométrie et Biologie Evolutive, Université Lyon 1, CNRS, Villeurbanne, France; University of Western Ontario, Canada

## Abstract

Early periods in Earth's history have seen a progressive increase in complexity of the ecosystems, but also dramatic crises decimating the biosphere. Such patterns are usually considered as large-scale changes among supra-specific groups, including morphological novelties, radiation, and extinctions. Nevertheless, in the same time, each species evolved by the way of micro-evolutionary processes, extended over millions of years into the evolution of lineages. How these two evolutionary scales interacted is a challenging issue because this requires bridging a gap between scales of observation and processes. The present study aims at transferring a typical macro-evolutionary approach, namely disparity analysis, to the study of fine-scale evolutionary variations in order to decipher what processes actually drove the dynamics of diversity at a micro-evolutionary level. The Late Frasnian to Late Famennian period was selected because it is punctuated by two major macro-evolutionary crises, as well as a progressive diversification of marine ecosystem. Disparity was estimated through this period on conodonts, tooth-like fossil remains of small eel-like predators that were part of the nektonic fauna. The study was focused on the emblematic genus of the period, *Palmatolepis*. Strikingly, both crises affected an already impoverished *Palmatolepis* disparity, increasing risks of random extinction. The major disparity signal rather emerged as a cycle of increase and decrease in disparity during the inter-crises period. The diversification shortly followed the first crisis and might correspond to an opportunistic occupation of empty ecological niche. The subsequent oriented shrinking in the morphospace occupation suggests that the ecological space available to *Palmatolepis* decreased through time, due to a combination of factors: deteriorating climate, expansion of competitors and predators. Disparity changes of *Palmatolepis* thus reflect changes in the structure of the ecological space itself, which was prone to evolve during this ancient period where modern ecosystems were progressively shaped.

## Introduction

The Paleozoic (-550 to -250 Myrs) is a challenging period for paleobiological studies because ecosystems were increasing in complexity through the appearance and radiation of different groups of organisms, progressively colonizing and even creating ecological niches (nekton in the marine realm, terrestrial habitat for plants and animals), up to building ecosystems close to those still existing today. These trends can be seen as emergent properties of supra-specific taxonomic units radiating, competing and going extinct [1], [2], [3], [4], [5]. These processes are commonly referred as “macro-evolution”. Yet, each species also evolved by the way of micro-evolutionary processes, extended over long period of time. Assessing the relationship between the patterns and processes acting at different evolutionary scales remains however difficult, because their study relies on different scales of analysis. Macro-evolutionary studies require large scale analyses of many taxa often pooled into high taxonomic units. In contrast, the analysis of fine-scale evolution within species, that could be referred as extended micro-evolution, requires focusing on a given taxon at a more restricted geographical and temporal scale. The present study aims at questioning this relation between different scales of processes by developing a fine-scale evolutionary approach in a context known for outstanding macro-evolutionary events. The Late Devonian is marked by two major biotic crises. The first one punctuated the Frasnian/Famennian (F/F) boundary. It had a particular dramatic impact on the biosphere, being considered as one of the “Big Five” mass extinctions in Earth's history [6]. The second one coincided with the Devonian/Carboniferous (D/C) boundary. It strongly impacted the biosphere as well, although less than the F/F crisis [7]. Because of these two outstanding events, most studies devoted to the Late Devonian focused on either the F/F or D/C boundary, questioning the processes triggering the mass extinctions and the patterns of post-crisis recovery [7], [8]. Independently of the crises, however, the Late Paleozoic was also characterized by long-term environmental trends [9], [10] and by an increase in complexity of ecosystems in the terrestrial [11] but also in the marine realm where the water column was progressively occupied by nektonic organisms [12].

In such macro-evolutionary studies, the dynamics of a species is often summarized by the range of its temporal occurrence, or even summed up with other relatives into large taxonomic groups. This hinders to address how micro-evolutionary processes interplayed with macro-evolutionary trends. The present study aims at filling this gap by performing a fine-scale evolutionary analysis along the Late Devonian, a period of *ca.* 15 myrs between the two successive F/F and D/C crises. We focused on conodont animals, small eel-like predators [13] that were part of the nektonic fauna increasing in richness through the Paleozoic period. Their fossil remains are tooth-like elements. In many modern organisms, shape variations in such traits involved in the feeding performance have been shown to trace variations in resource exploitation [14], [15], [16]. This ecomorphological approach was applied to the shape variation observed within the conodont genus *Palmatolepis*, the most abundant conodont of the Late Devonian, disappearing during the D/C crisis. Using geometric morphometrics to quantify the shape of the tooth-like elements, the pattern of diversification of *Palmatolepis* was assessed through time. Questions addressed included (1) did the F/F crisis impoverish the morphological diversity within *Palmatolepis*, as expected based on macro-evolutionary patterns marking this mass extinction? (2) Was the screening of the crises random or not regarding ecomorphs within *Palmatolepis*? (3) Did *Palmatolepis* diversified subsequently to the F/F crisis in a random or selective way? (4) What is the timing of this diversification subsequent to a crisis? Was it mostly conditioned by the catastrophic mass extinction events, with a pattern of post-crisis diversification followed by a stable disparity, until facing the next mass extinction event? Alternatively, was the diversification dynamics mostly driven by long term climatic and/or ecological trends developing between the two crises?

## Materials and Methods

### Material

#### Stratigraphic background

**Figure 1:**
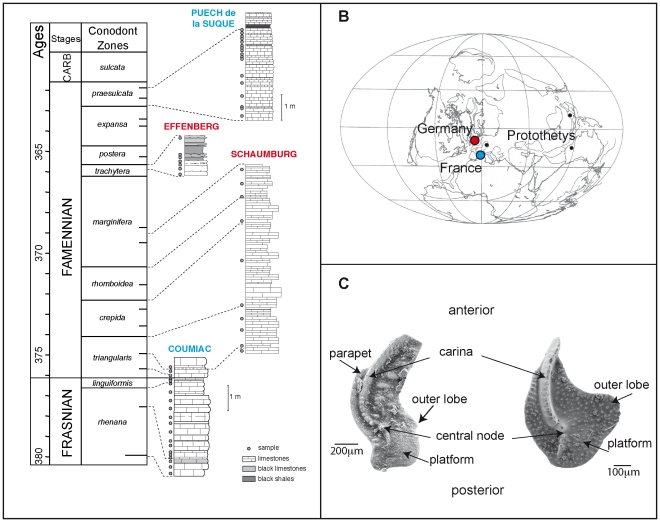
Temporal and geographical sampling, and terminology on conodont elements. (A) Timescale, composite section along the end Frasnian and the Famennian and stratigraphic log of the studied sections. Absolute ages after [24] and conodont zones after [77]. Note that the *postera* and *expansa* zones are not sampled. Along the stratigraphic log of each section, dots represent the sampled levels. Abbreviations: E = Early, M = Middle, L = Late. (B) Paleogeographic map [19] of the Famennian. Circles = location of the French (blue circle) and German (red circle) sections contributing to the composite section. Black dots = location of the sections that delivered additional sampling of *Palmatolepis (Palmatolepis) linguiformis*. (C) Illustration of *Palmatolepis* platform elements, with the terminology of the morphological features used in taxonomy. To the left a specimen of *Pa. (Palmatolepis) rugosa* (*trachytera* zone). To the right a specimen of *Pa. (Manticolepis) rotunda* (*rhenana* zone).

A composite record has been established based on samples from four sections, two located in the Montagne Noire in Southern France (Coumiac and Puech de la Suque) and two located in the Rhenish Massif in Germany (Schaumburg and Effenberg) (Fig. 1A–B). All the levels which delivered conodont elements were of limestone composition [17]. The local differences in geological setting have been shown to have little influence on the morphometric characteristics of the conodont assemblage [18].

According to paleogeographic reconstructions [19], all studied locations were situated in tropical to subtropical paleolatitudes between 10 and 30° southern latitude (Fig. 1B). The Montagne Noire and the Rhenish Massif were separated by the Protothetys but the distance between these two areas was approximately the same as today.

The Frasnian has only been recognized in Coumiac (France) where the last two zones (*rhenana* and *linguiformis*) of this period are represented [20].

The index species for the *linguiformis* zone is extremely rare in Coumiac where it has been only documented by a single conodont element [21]. This species is, however, the sole representent of the subgenus *Palmatolepis (Palmatolepis)* at that time. In order to provide a reliable estimate of its contribution to the morphological variance within *Palmatolepis*, its sampling has been increased by including *Palmatolepis (Palmatolepis) linguiformis* elements from other localities around the Protothetys [20].

Concerning the beginning of the Famennian, the first zone (*triangularis*) has been documented in Coumiac [20], [22] and in Schaumburg. In order to trace fine scale temporal variations around the key period of the Late Frasnian crisis, both the *rhenana* and the *triangularis* zones have been subdivided into subzones. Only the latest of the *triangularis* subzone is documented in Schaumburg.

The three following zones of the Famennian (*crepida*, *rhomboidea* and *marginifera*) have been documented in Schaumburg.

Samples from the Effenberg section document the *trachytera* zone [17]. The *praesulcata* zone has been sampled in the Puech de la Suque section [23].

Unfortunately, two Famennian conodont zones (*postera* and *expansa*) are missing in this study between *trachytera* and *praesulcata*.

Overall, the samples range in age from the end Frasnian (376.1±3.6 Ma) to the end-Famennian (360.7±2.7 Ma) [24].

#### Conodont elements for morphometric analyses

Each rock sample along the composite section was dissolved in formic acid (10%) and rinsed through two sieves. The fraction between 100 µm and 1 mm was then picked for all conodont elements.

The conodont animal had a complex feeding apparatus composed of several elements. In the case of *Palmatolepis*, the apparatus was composed of seven distinct elements (with right and left elements for six of them). The morphometric study focused on the so-called P1 platform element [25] (Fig. 1C) for several reasons. First, this element was the most robust within the former apparatus and therefore with the highest preservation potential. Second, P1 elements display very characteristic morphological traits that make them crucial for the description of the morphological and taxonomical diversity within *Palmatolepis* [26]. Many of these characteristics are expressed by variations of the platform shape that can be adequately described by a morphometric analysis [27], [28], [29]. For morphometric analyses, all unbroken platform (P1) elements of the genus *Palmatolepis* (*Pa.*) were picked over the composite section and measured, e.g. 5171 elements in total.

**Figure 2:**
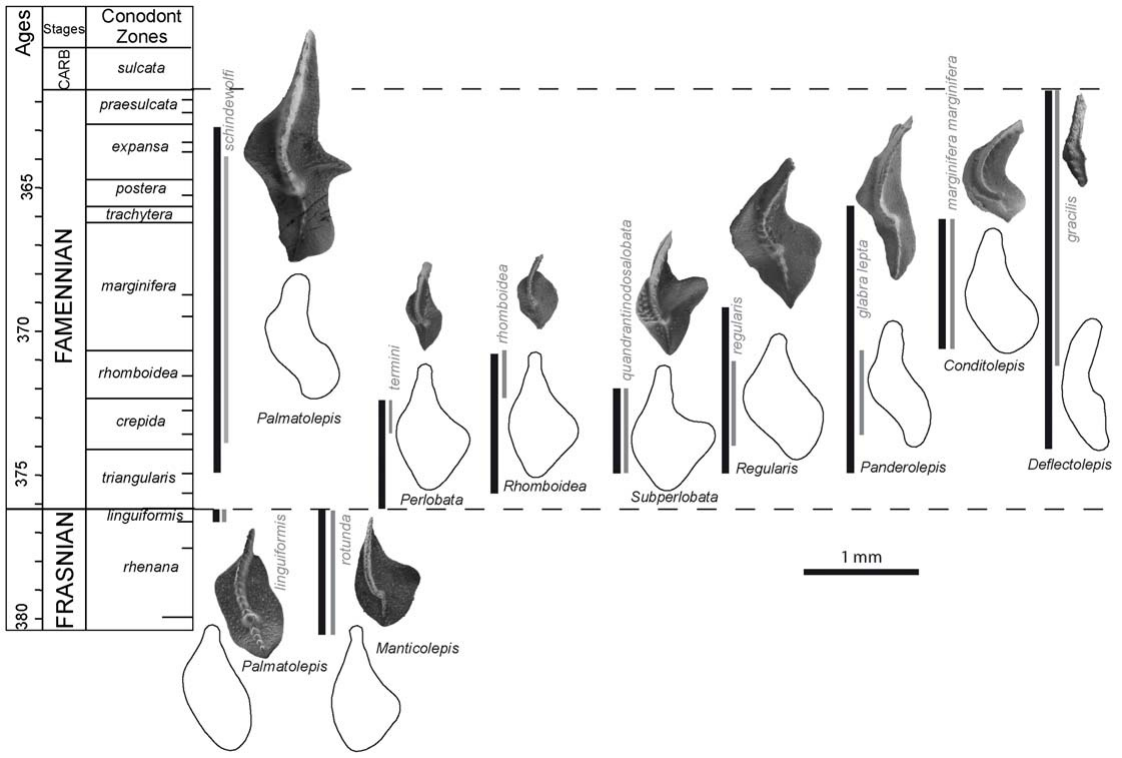
Temporal distribution and illustration of the *Palmatolepis* groups (end Frasnian to end Famennian). Each group is illustrated by a characteristic specimen and the average reconstructed outline. Vertical black bars represent the temporal extension of the groups, grey bars the temporal distribution of the (sub)species of the illustrated specimen. The horizontal dotted lines represent the F/F and the D/C crises.

The morphological diversity within *Palmatolepis* elements through time led to the description of numerous species and even subspecies. These (sub)species delivered valuable stratigraphic data [30] but their meaning as corresponding to former biological species is debated [26]. They especially present the problem to let a large proportion (up to 90% [31]) of the specimens unidentified and thus not included in the estimation of the morphological variance. The subgenus has been proposed to be closer to what former conodont species might have been, by including a component of geographic and temporal variance that is otherwise split into separate, static units [18], [32], [33]. To tackle this problem regarding the basic unit for estimating shape variations, three complementary levels have been considered. (1) All *Palmatolepis* elements have been considered independently of any further taxonomic identification. (2) As a conservative approach, to be as close as possible to the current conodont taxonomy, *Palmatolepis* elements were attributed to the species (and subspecies whenever existing) level, a part of the samples being then let aside as non-identified specimens. (3) *Palmatolepis* elements were attributed to larger “groups” of specimens sharing a range of general characters (Fig. 2).

Five of these groups corresponded to taxonomically described subgenera: *Manticolepis*, *Palmatolepis*, *Deflectolepis*, *Panderolepis* and *Conditolepis* [34], [35], [36], [37]. The four other groups were tentatively proposed based on the literature [38], [39].

The *Manticolepis* group was typical of the Frasnian period. It is characterized by elements with an asymmetrical and large platform. Elements are as broad as long. The outer lobe is always present and prominent. The carina is straight. The central node has the same size than other nodes of the carina. In our study, this group is documented by the species *rhenana, rotunda, subrecta*, and *hassi*.
The *Palmatolepis* group had both Frasnian and Famennian representatives. They share a sinuous carina, and a small outer lobe. During the Famennian, the representatives of this group are the largest of all *Palmatolepis*. They present a distinct secondary carina on the outer lobe. In this study, the representatives are the Frasnian *linguiformis* and the Famennian *perlobata schindewolfi* and *rugosa rugosa*.
The *Panderolepis* group is characterized by elements with slender, triangular and smooth platforms. The platform does not present an outer lobe but has a parapet on the inner side, anterior to the central node. The carina is sigmoidal. This group is represented by the species *tenuipunctata* and *glabra* with three subspecies: *glabra lepta*, *glabra pectinata* and *glabra distorta*.
The *Conditolepis* group is characterized by elements without outer lobe, and with round platforms with a sharp parapet and with a carina developed posterior of the large central node. The anterior part of the carina is more curved than for any other groups. It is represented here by the species *marginifera* with two subspecies *marginifera marginifera* and *marginifera inflexoidea*.
The *Deflectolepis* group is characterized by small, narrow elements with a smooth platform well-developed posterior to the central node. The outline of the platform is elongate. *Deflectolepis* is always slender than other groups. The carina is straight. The representatives included in this study are the two species with a single subspecies *gracilis gracilis* and *minuta minuta*.
The *Subperlobata* group includes elements with a sigmoidal carina and a central node more developed than the other nodes of the carina. At the posterior edge of the element, the platform is triangular but asymmetrical with regards to the posterior carina. The outer lobe is present and forms an acute angle with the blade. The platform is not smooth but punctuated of nodes and/or ornamentation. It is documented in our samples by the four species *quadrantinodosalobata*, *praeterita*, *spathula* and *lobicornis*.
The *Perlobata* group corresponds to elements with a triangular and smooth platform, and a free blade with a straight carina. The elements are arched, the posterior edge being tilted upwards. The node is at the center of the blade and is more developed than the other nodes along the carina. An outer lobe is present. In the study, this group is documented by the species *praetriangularis, triangularis, crepida* and *termini*.
The *Rhomboidea* group is characterized by elements with a diamond-shape and smooth platform outline. They are always smaller than elements of the other groups. The carina is sigmoidal, and not developed posterior to the central node. It is sampled here by the species *delicatula*, *clarki*, *protorhomboidea* and *rhomboidea*.
The *Regularis* group includes sigmoidal elements with both sides of the platform parallel to the carina. The upper platform is smooth. It is represented here by the species *regularis*.

The numbers of conodont elements included in the morphometric analysis are detailed by group, by (sub)species and by (sub)zones in the Table 1.

**Table 1:** Sampling of the different groups and (sub)species through end-Frasnian and Famennian.

	Zones	Early *rhenana*	Late *rhenana*	*linguiformis*	Early *triang*.	Middle *triangularis*	Late *triang*.	*crepida*	*rhomboidea*	*marginifera*	*trachytera*	*praesulcata*
Groups	(sub)species											
Manticolepis	*rotunda*	20	10									
	*subrecta*	11	16									
	*rhenana*		6									
	*gigas*		3									
	*indet*	545	1698	220								
Palmatolepis	*linguiformis*			59								
	*perlobata schindewolfi*						55	49		49	33	
	*rugosa rugosa*										7	
Perlobata	*triangularis*				10	84	51	16				
	*praetriangularis*				25	34	62					
	*crepida*							18				
	*termini*							22				
Rhomboidea	*clarki*					12	21					
	*delicatula*					22	12					
	*protorhomboidea*					3	12					
	*rhomboidea*								43			
Regularis	*regularis*						4	23		21		
Subperlobata	*spathula*						46					
	*lobicormis*						2	5				
	*praeterita*							11				
	*quadrantinodosalobata*							66				
Panderolepis	*tenuipunctata*						21	43				
	*glabra lepta*							8	81	196	21	
	*glabra pectinata*								35	32		
	*glabra distorta*									30		
Conditolepis	*marginifera marginifera*									218		
	*marginifera inflexoidea*									5		
Deflectolepis	*minuta*							39	40	34	18	
	*gracilis*									11	88	520
indet					16	28	147	60	20	54		
Total/zone		576	1733	279	51	183	433	311	268	650	167	520

Number of *Palmatolepis* elements measured for the morphometric analysis, detailed by stratigraphic zones (columns) and taxonomic units (rows). Groups and (sub) species are presented in order of their first occurrence in the successive stratigraphic zones.

### Methods

#### Morphometric Analysis

The two-dimensional analysis of the platform outline has been shown to efficiently describe geographic, temporal, and taxonomic variations of *Palmatolepis* throughout its record (e.g. [27], [28], [29]). Because conodont animals had a bilateral symmetry, both right and left elements were found in an assemblage. Left elements were subjected to a mirror transformation and measured as right elements. The two-dimensional outline of each conodont was automatically digitised using an image analyser (Optimas). For each conodont element, *x*- and *y*-coordinates of 64 points were sampled at equally spaced intervals along the outline. The starting point was defined at the tip of the carina (Fig. 1C).

The variation of the distance of each point to the center of the outline as a function of the cumulative distance along the outline was decomposed into a sum of trigonometric functions of decreasing wavelengths (harmonics), each being weighted by two Fourier coefficients (FCs). The zero harmonic, proportional to the outline size, was used to standardize all other FCs so they represent shape variables only. Considering the first eleven harmonics has been shown to provide a satisfying compromise between the number of variables and the amount of shape information (e.g. [29]). One of the advantages of the Fourier functions is that an outline can be reconstructed by successive additions of the different harmonics from any set of Fourier coefficients, allowing a visualization of the shape changes [40], [41].

**Figure 3:**
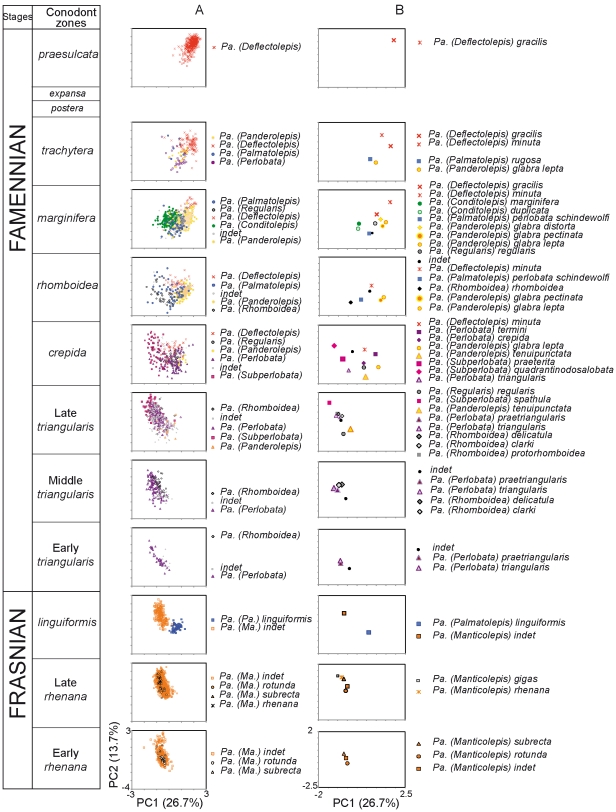
Occupation of the *Palmatolepis* morphospace through the end Frasnian and the Famennian. The morphospace is defined by the first two axes of a principal component analysis on the Fourier coefficients of the platform outline. The representation has been split by temporal zones. (A) Total variation (each dot corresponds to a single specimen). Symbols correspond to the morphological groups illustrated on the Fig. 2. (B) Variation among (sub)species (each dot corresponds to the average value of the corresponding taxonomic unit). Colours correspond to morphological groups.

**Figure 4:**
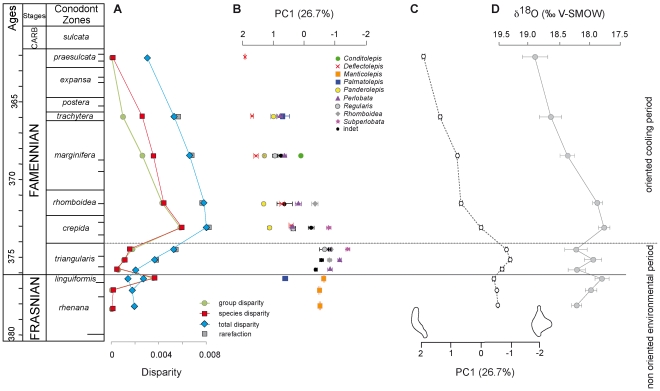
End Frasnian to end Famennian variations in *Palmatolepis* disparity and morphology, and paleoenvironment. (A) Temporal variations in *Palmatolepis* disparity. Blue diamonds = total disparity (for the *linguiformis* zone, two values are provided: including additional *Pa. (Pa.) linguiformis* [full line] or relying on Coumiac sampling only [dotted line]). Red squares = (sub)species disparity. Green circles = group disparity. Disparity was estimated as the variance among units (elements, (sub)species or groups), i.e. the sum of the variance of the Fourier coefficients. Robustness of the total disparity estimate to variations in sampling has been evaluated by a random rarefaction procedure, each sample being randomly subsampled 10 times to the lowest sample size (51 elements). Error bars (95% confidence interval [CI]) are represented but are masked by the symbol of the mean value of the rarefied samples (grey squares). (B) Morphometric variation of the different *Palmatolepis* groups through time. The first axis (PC1) of the morphospace has been considered as synthetic shape axis. Average value +/−95% CI has been represented for each group. (C) Morphometric variation of the total assemblage through time, represented by the average value +/−95% CI per zone of all elements on the synthetic shape axis (PC1). Reconstructed outlines visualize the shape change along PC1, corresponding to the presence (negative values) or absence (positive values) of a lobe. (D) Variation in sea-surface temperature per zone based on conodont apatite ∂^18^O (data from [10]), +/−95% CI. The solid line corresponds to the Frasnian/Famennian boundary. The dashed line represents the separation between two periods characterized by different environmental conditions: at the base an overall warm period with non-oriented fluctuations, followed by an oriented cooling trend.

Synthetic shape axes were extracted using a principal component analysis on the correlation matrix of the FCs. This method has been chosen because it is independent of any *a priori* taxonomic attribution. The first two principal components define a morphospace on which the range of shape variation can be represented. The temporal variation in the occupation of this morphospace has been visualised by plotting each zone separately (Fig. 3). Different levels of variation were considered: the variation among all elements present at a given time (Fig. 4A), or at a higher taxonomic level, among (sub)species and among groups present (Fig. 4B).

#### Morphological disparity

The morphological disparity quantifies the diversity of shape among a set of units that can be species, higher taxonomic entities, or more rarely single specimens.

Several estimates can be considered. The most frequently used are (1) the range encompassed by units along axes of a morphospace, or (2) the inter-unit variance [3], [42]. Because it is independent of the computation of synthetic axes, we have selected the second disparity estimate, namely the morphological variance (i.e. the sum of the variance of the eleven FCs) among units. Using this estimate, disparity has been evaluated at three taxonomic levels: considering as unit each single element (total disparity), considering as unit each (sub)species (“species” disparity) or each group (group disparity).

Estimation of the morphological variance can be affected by the number of specimens available [43]. In the present study, differences in the number of conodont elements available in each zone are dramatic: from 51 in the Early *triangularis* zone to 1733 in the Late *rhenana* zone (see Table 1). This might affect the estimation of the morphological variance (hence the total disparity). We thus used a rarefaction procedure to test this influence. We artificially decreased the sampling of all other zones to the smallest sample size (51 elements), using a random choice within the sample available. To estimate the error related to this random sampling, we repeated the procedure ten times. This provided an estimate of the variance in the estimation of the total disparity in all zones except for the Early *triangularis* zone.

#### Relationships between morphometrics, disparity, and paleoenvironmental proxies

We obtained for each zone three estimates of disparity (total, by (sub)species and by group). Scores on the first principal axis were considered as a synthetic shape variable and provided estimates of shape variations along the record, either for all elements (total shape) or for (sub)species and for each group separately.

Disparity and shape variations were further compared with a paleotemperature proxy, represented by a composite record of ∂^18^O values of conodont phosphate [10]. Note that as for the conodont samples, all geochemical data were obtained from tropical to subtropical palaeolatitude paleolocations.

Relationships among morphological estimates, and between morphological and paleoenvironmental proxies were investigated. Several complementary tests were used. (1) Linear regressions were used to confront paleoenvironmental data (considered as independent variables) to morphological variables (dependent variables). Such an approach compares the raw data and the amount of variation between two successive samples impacts the regression. (2) Spearman-rank order correlations were used to further test the relationship between the variables. Values of each variable are ranked and these ranks are then compared among variables. This limits the impacts of extreme shifts that can be for instance due to a long time elapsed between two successive samples. (3) A correlation of paleoenvironmental and morphological variables could be due to the fact they both vary concomitantly through time. The data were thus detrended by considering the first difference (difference between two successive values along a time-series) and thereafter compared using the above mentioned procedures (linear regression and Spearman rank-order correlation).

## Results

### Temporal variations in the morphospace

The first two principal axes (PC1 = 26.7% and PC2 = 13.7% of the total variance) defined a morphospace on which the distribution of all *Palmatolepis* platform elements has been represented through time (Fig. 3A).

Towards the end of the Frasnian (*rhenana* zone), *Palmatolepis* elements were tightly clustered in the part of the morphospace corresponding to negative PC1 and positive PC2 scores. This cluster corresponded to conodonts attributed to the *Manticolepis* group. A limited diversification occurred during the latest zone of the Frasnian (*linguiformis* zone). A cluster occupied a formerly empty zone of the morphospace, towards slightly positive PC1 values. This cluster corresponded to the occurrence of the *Palmatolepis* group.

The earliest Famennian, just after the Frasnian/Famennian crisis (Early *triangularis* zone), was marked by a retraction of the morphological range towards the zone occupied during the *rhenana* zone. This corresponded to the occurrence of conodonts from the *Perlobata* and *Subperlobata* groups.

During the latest part of the *triangularis* zone, a reoccupation of the range formerly occupied by *Palmatolepis* was initiated. The *crepida* zone was characterized by a wide occupation of the morphospace, with an extension towards positive PC1 values. This was concomitant with the occurrence of the *Regularis* and *Deflectolepis* groups. Starting during the *rhomboidea* zone, but more extreme during the *marginifera* zone, a shift occurred towards a dominance of elements with positive PC1 values. A median range was still occupied by the *Conditolepis* group, whereas elements of the *Panderolepis* group clustered towards positive PC1 and median PC2 values, and *Deflectolepis* elements clustered towards extreme positive PC1 and PC2 values.

During the *trachytera* zone, the range occupied shrinked towards positive PC1 only, a trend that culminated during the latest zone of the Famennian (*praesulcata*). *Deflectolepis* elements occupied then a limited zone of the morphospace, towards positive PC1 and PC2 values. This mirrored the situation that occurred during the end of the Frasnian, but with a cluster of elements in a completely different zone of the morphospace.

### From variations among elements to species and groups

Beyond these trends that corresponded to variations in the distribution of all *Palmatolepis* elements, (sub)species have been identified within groups. We investigated visually whether these units had a morphological coherence (Fig. 3A and B). Indeed, each group corresponded to a given zone of the morphospace (Fig. 3A), despite some overlap, especially during the highly diversified periods (*crepida*, *rhomboidea* and *marginifera* zones). Accordingly, (sub)species of a given group tended to cluster together in the morphospace (Fig. 3B). Using the inverse Fourier transform method, average shape of each group was visualised (Fig. 2). They illustrated how the morphological characteristics that served to define each group were mirrored on the morphometric description of the outline.

### Patterns of disparity

Patterns of disparity observed among the total assemblages of elements, among (sub)species and among groups were remarkably similar (Fig. 4A). Starting from a low disparity during the *rhenana* zone, a momentary increase occurred during the *linguiformis* zone due to the co-occurrence of two groups (*Manticolepis* and *Palmatolepis*). Noted that the increase in total disparity was slightly emphasized by the supplementary *Pa. (Pa.) linguiformis* specimens that had been added to the original sample. Disparity was back to a very low level just after the F/F crisis (lower *triangularis* zone), started increasing from the middle *triangularis* onward, up to culminate during the *crepida* zone. From this maximum it gradually decreased down to levels during the end Famennian (*praesulcata* zone) as low as during the end Frasnian.

Disparity estimates based on (sub)species and based on groups provided highly similar results (linear correlation, R^2^ = 0.93, P<0.001). This result suggested that splitting groups in different species did not explain much of the morphological variance and it reinforced the use of the groups as relevant biological units. Considering the disparity among the total assemblage of specimens is a seldom used approach, because this estimate may vary depending on the relative abundance of the different taxonomic units. Our results showed that this approach provided the same trends as the disparity among species or groups (total *vs.* species disparity: R^2^ = 0.73, P = 0.001; total vs. group disparity: R^2^ = 0.64, P = 0.003). Disparity estimate based on the total assemblage of specimens appeared as quite robust to sampling issues as shown by the estimates based on rarefied samples that were similar to those based on the total sample. Furthermore variance among replicates of the rarefaction procedure was so reduced that error bars corresponding to a 95% standard error were not visible on curves (Fig. 4A). This approach may thus constitute a valuable alternative to estimate disparity levels in groups where taxonomic units are debated and difficult to identify with a reliable biological meaning [26].

### Patterns of shape variation

Since the distribution in the morphospace showed a progressive shift in the zones that were occupied, we investigated how this corresponded to shape changes through time. Once again, two levels of variation were considered: trends per groups (Fig. 4B), and the total trend corresponding to the average shape of all *Palmatolepis* elements at a given time (Fig. 4C).

Each group was characterized by a given range along PC1 (e.g *Manticolepis* around -1, *Panderolepis* around 1.2, *Deflectolepis* around 1.6), supporting its definition as separate unit. Beyond this morphological coherence of the groups, the range they occupied often varied through time. *Perlobata* as well as *Regularis* displayed temporal variations, starting from negative PC1 close to those of the Frasnian *Manticolepis* to evolve towards positive scores similar to typical Famennian groups such as *Panderolepis*. Similarly, *Deflectolepis* displayed a trend towards more extreme shapes along the Famennian.

Variation in the total average shape were further considered along PC1 (Fig. 4C). Overall, this axis opposed platform elements with a developed lobe (negative PC1) and elements without a lobe (positive PC1). The average shape per level included and summarized different sources of variation: succession of groups with different characteristic shapes, temporal variations within these groups, and changes in the relative abundance between the groups (Fig. 4C). Despite this composite information, coherent temporal trends emerged. Total average shape was quite stable during the end Frasnian. A slight shift occurred during the *triangularis* zone towards conodonts with a more developed platform, but the average shape was still quite stable. A trend of regression of the lobe (increase along PC1) started in *crepida* zone and continued up to the latest zone of the Famennian (*praesulcata*).

### Comparison with paleoenvironmental trends

Oxygen isotope data [10] provided a proxy for temperature variations throughout the period. Two main periods emerged regarding these paleoenvironmental variations (Fig. 4D): an overall warm period with short-term fluctuations (from the end Frasnian to the end of the *triangularis* zone) followed by a trend of decreasing temperature until the end of the Famennian.

In order to investigate a possible impact of these variations on the disparity pattern and morphological evolution of *Palmatolepis*, we compared the morphological parameters (average shape and disparity estimates) with this paleotemperature proxy. Combining all tests (linear and rank-order correlations on raw and detrended data; Table 2), the major patterns emerging can be summarized as follow. (1) Over the whole period, shape and disparity tended to co-vary with the paleoenvironmental proxy. (2) Such correlations failed to be evidenced during the first part of the record, corresponding to non-oriented environmental fluctuations. (3) In contrast, variations in shape and disparity are related to environmental changes during the second part of the record, corresponding to the cooling trend.

**Table 2:** Relationship between *Palmatolepis* shape and disparity, and paleoenvironmental variations.

		Raw								Detrend					
Record	∂^18^O	N	R_lin_	P_lin_	P_lin_*	R_SRO_	P_SRO_	P_SRO_*	N	R_lin_	P_lin_	P_lin_*	R_SRO_	P_SRO_	P_SRO_*
Total	PC1	11	0.700	**0.016**	**0.013**	0.418	0.201	0.133	10	−0.107	0.769	0.953	−0.285	0.425	0.934
	Disp_Tot_	11	−0.152	0.655	0.125	−0.082	0.811	0.235	10	−0.699	**0.025**	**0.010**	−0.685	**0.029**	0.055
	Disp_Gp_	11	−0.572	0.066		−0.510	0.109		10	−0.769	**0.009**		−0.717	**0.020**	
	Disp_Sp_	11	−0.446	0.169		−0.428	0.190		10	−0.764	**0.010**		−0.636	**0.045**	
Part 1	PC1	6	−0.206	0.695	0.837	−0.423	0.355	0.714	5	−0.072	0.908	0.246	−0.100	0.783	0.450
	Disp_Tot_	6	0.064	0.904	0.680	0.143	0.714	0.933	5	−0.330	0.588	0.450	−0.600	0.233	0.683
	Disp_Gp_	6	−0.502	0.310		−0.320	0.650		5	−0.549	0.337		−0.200	0.683	
	Disp_Sp_	6	−0.595	0.213		−0.232	0.650		5	−0.642	0.243		−0.500	0.450	
Part 2	PC1	5	0.953	**0.012**		1.000	**0.017**		5						
	Disp_Tot_	5	−0.961	**0.009**		−1.000	**0.017**		5						
	Disp_Gp_	5	−0.994	**0.006**		−1.000	**0.017**		5						
	Disp_Sp_	5	−0.943	**0.016**		−1.000	**0.017**		5						

Correlations between the paleoenvironmental proxy (∂^18^O) and *Palmatolepis* mean shape (PC1), total disparity (Disp_Tot_), group disparity (Disp_Gp_) and (sub)species disparity (Disp_Sp_) were calculated using linear regressions (lin) and Spearman rank-order correlation (SRO). For both, the number of items (N), the coefficient of correlation (R) and the probability of the correlation (P) are provided. A star (*) indicates values calculated without the specimens of *Pa. (Pa.) linguiformis* added to the one of Coumiac. To the left, values based on raw data; to the right, values based on detrended data (first order difference, i.e. differences between successive samples).

The correlations have been estimated for the whole record (Total), its first part corresponding to non-oriented climatic fluctuations (Part 1, from *rhenana* to *triangularis*), and on its second part marked by a cooling trend (Part 2, from *crepida* to *praesulcata*).

## Discussion

Disparity describes how organisms are distributed in a morphological space. A first component in structuring this distribution is the phylogenetic history of the groups. Closely related species, sharing numerous genes and developmental pathways, usually display close morphologies whereas an increased phylogenetic divergence leads to an increased morphological differentiation (e.g. [44], [45], [46]). Yet, such a passive diffusion in a morphospace appears to be slow when compared to shifts due to morphological innovations related to adaptation to new ecological niches [46], [47]. As a consequence, taxa sharing similar ecology will tend to display similar morphologies, making the occupation of the morphospace a signature of the resource partitioning in the ecological space.

Assessing patterns of disparity through time can thus bring precious insights into the processes driving diversification – or extinction. The Late Devonian was punctuated by two outstanding biotic crises, and the first step was thus to consider how these crises impacted the small-scale diversity within our model organism, the conodont *Palmatolepis*. The interpretation of the disparity patterns for this fossil group was confronted to the problem of identifying the relevant level of variation to estimate morphological variance. The congruence of all disparity patterns, estimated on the set of all specimens, on the stratigraphic species, or on larger groups supposed to be closer to what the biological species might have been [26], makes us confident that beyond such debates, robust trends emerged as a basis for fine-scale evolutionary and ecological interpretations.

### Impact of major biotic crises at a fine evolutionary scale

Both the F/F and the D/C crises were characterized by environmental perturbations similar in their nature [7] including rapid sea-level fluctuations [48] and abrupt changes in sea-surface temperatures [10], [49], [50]. Their differential impact on the biosphere was apparently related to differences in the timing and magnitude of the environmental perturbations [7], [51], [52]. Reverse to what occurred to many other groups, *Palmatolepis* survived the F/F but went extinct during the D/C crisis.

A candidate explanation of this differential survival is that the set of related species constituting *Palmatolepis* did not confront each crisis with the same potential of survival. Indeed, pre-F/F and pre-D/C *Palmatolepis* occupied opposed areas of the morphological space, suggesting adaptations to very different ecological conditions. Shortly before their extinction during the D/C crisis, *Palmatolepis* conodonts did only display elements with extremely reduced platform. During the F/F crisis, the group going extinct (represented by *Pa. (Pa.) linguiformis*) also tended to present a reduced platform. This trait might thus have constituted a factor of increased risk of extinction. Although the conodont diet is still largely unresolved, a developed platform argues for the ability to handle robust food, whereas a reduced platform would better match the consumption of soft food. The ability to exploit a variety of resources, including robust prey, might have been a selective advantage at a time when resources were anyway decimated by the global crisis.

Another, not mutually exclusive factor increasing risk of extinction, is to face the crisis with an already impoverished diversity. Then, extinction is more probable even when occurring at random [3], [53], [54]. Remarkably, *Palmatolepis* presented two cases matching this scenario, since its disparity was at its lowest just before both the F/F and the D/C crisis. Hence, the typical macro-evolutionary pattern of depletion in diversity marking major crises cannot be recognised in the fine-scale evolutionary pattern of *Palmatolepis*. Far from being highly diversified before, and impoverished by the crises, it faced them with an – unfavourable – low diversity.

Thus, at this scale of disparity, the driving force of disparity does not seem to be the major, punctual events of the crises. More progressive processes seem to have shaped conodont disparity during the period between the crises. Such processes went largely unnoticed because that period deserved little interest among researchers due to the overall stability of fauna in terms of diversity.

### An inter-crisis cycle of increase and decrease in disparity

The expected pattern of impoverished disparity consecutive to the major event of a biotic crisis was not documented along the record of *Palmatolepis* morphological evolution. In contrast, a progressive diversification occurred shortly after the crisis. This pattern matches the expectation of a “recovery period” during which the impoverished ecosystem is increasing again in complexity. The crisis, by decimating many groups, is supposed to have let vacant many ecological niches that the post-crisis organisms progressively re-occupied [55], [56], [57], [58], [59], [60]. The pattern of diversification within *Palmatolepis* matched, at this fine evolutionary scale, the expected macro-evolutionary pattern of recovery. The diversification occurred in few million years, along the first zone consecutive to the crisis. It also occurred in a selective manner, with an oriented trend corresponding to the progressive occupation of morphospace areas corresponding to slender elements with a more and more reduced platform. Based on an ecomorphological interpretation of these variations, this suggests a diversification of the food resources exploited by *Palmatolepis*, a process favoured by the decimation of the ecosystem that occurred shortly before, towards the consumption of other, presumably softer preys.

A more surprising feature is that this diversification did not continue until the next major biotic crisis punctuating the D/C boundary. It did not even reach a plateau of stable disparity, associated with a subsequent increase in diversity: such a pattern is the sign of rapid morphological innovations allowing the conquest of new ecological niches, followed as a subsequent step by a finer resource partitioning between related species [16]. Rather, disparity started a gradual decrease soon after reaching its maximum. This pattern cannot be related to any catastrophic mass extinction events and rather point to the importance of other, more discrete factors in driving disparity trends in the inter-crisis period. The decrease in disparity was further selective, with a progressive switch towards a dominance of platform-reduced morphologies to the detriment of shapes with a well-developed platform that were characteristics of the beginning of the record.

This decrease in disparity appears to be related to the cooling trend characterizing the time period. This may be due to coincident trends without causal relationship. Yet, *Palmatolepis* was characterized by warm-water preferences, being restricted to tropical and sub-tropical areas [39], [61], [62]. It is thus likely that the cooling trend was unfavourable to this taxon. The selectivity of the disparity decrease further suggests that resources available to *Palmatolepis* were shrinking towards the end of the Devonian. This may be due to a decrease in primary productivity itself, but the composition and dynamics of the primary producers, and even more the feedbacks between the carbon cycle and the climate change, are far from being understood in this deep past [63], [64], [65]. More simply, *Palmatolepis* might have been deprived of access to resources it formerly consumed by changes in the ecosystem itself. First, each group had its own timing of post-crisis recovery, and possibly *Palmatolepis* acted as an opportunistic taxon rapidly colonizing empty niches but thereafter outcompeted by more specialist groups [66]. In support of this interpretation, *Palmatolepis* has been suggested to be an opportunistic taxa investing much in early reproduction and achieving a rapid turnover [67], traits prone to rapidly colonize perturbed environments. Candidate competitors included *Bispathodus*, another conodont genus that increased in diversity along the Famennian to represent the dominant component of the conodont fauna towards the end of the period [23], [30], [68]. Its increase in absolute abundance, however, did not fully compensate the decrease in *Palmatolepis* [32], suggesting that it was not the sole competitor involved.

Beyond the dynamics of post-crisis recovery, the evolution of *Palmatolepis* may have interfered with processes occurring at a longer time pace. During the Late Paleozoic, the ecosystems were increasing in complexity because of the diversification of major groups. This is the case of ammonoids and chondrychthians [69], [70] that were competing with conodonts for resources [70], [71]. This large-scale process of diversification did not only involve competitors, but also predators such as sharks and fishes [72], [73], thus increasing selective pressures on conodonts even beyond access to resources.

### Interplay of evolutionary processes at different scales and paces

The selective impoverishment of *Palmatolepis* disparity could thus be the result of an unfavourable combination: deteriorating climate, diversification of predators, and increasing competition at different paces, from the F/F recovery to long-term increase in complexity of the ecosystems. All could have contributed to its decrease in diversity and disparity predating the D/C crisis. Without being the direct cause of *Palmatolepis* extinction, all of these factors could have thus increased the risk of extinction; similar dynamics seems probable just before the F/F crisis, although this part of the record is not documented here. In this context, the major events constituted by the crises do not emerge as the key factors driving disparity dynamics within *Palmatolepis*. Rather, the progressive change in the structure of the ecological space seems to have conditioned the diversification and subsequent impoverishment of *Palmatolepis*.

This interplay of evolutionary and ecological processes is emerging as crucial to understand the dynamics of perturbed ecosystems [74], [75]. The dynamics of *Palmatolepis* illustrates this interaction in the deep past. Understanding the emerging patterns of biodiversity is difficult because of such subtle interplay between ranges of processes that were usually considered separately, and even more by the interaction of different scales and paces of processes. *Palmatolepis* disparity illustrates this intermingling of scales. It appears as the combined product of long-term trends in ecosystem complexity, middle-term trends in climate and dynamics of related groups, and even short-term evolutionary trends within each group composing *Palmatolepis*. Indeed, several *Palmatolepis* groups displayed oriented trends that mirrored, within each lineage, the trend followed by the genus as a whole. Fine-scale evolutionary analyses also demonstrated the ability of *Palmatolepis* species to track and adapt rapid temperature changes during the F/F crisis [20], [76]. Further integrative studies attempting to combine paleoenvironmental, paleoecological and evolutionary studies over intermediate time-scales will be necessary to decipher how evolutionary and ecological responses over short-time scale interplay and condition the mid- to long-term variation of the ecosystem, and contribute to an emergent macro-evolutionary signal.
